# Maximum depth sequencing reveals an ON/OFF replication slippage switch and apparent in vivo selection for *bifidobacterial* pilus expression

**DOI:** 10.1038/s41598-022-13668-2

**Published:** 2022-06-10

**Authors:** Christophe Penno, Mary O’Connell Motherway, Yuan Fu, Virag Sharma, Fiona Crispie, Paul D. Cotter, Benoit Houeix, Lokesh Joshi, Francesca Bottacini, Aoife O’Dwyer, Gary Loughran, John F. Atkins, Douwe van Sinderen

**Affiliations:** 1 ECOBIO, Université Rennes 1, 35042 Rennes, France; 2 School of Microbiology, University College Cork, Cork, T12 YT57 Ireland; 3 APC Microbiome Ireland, University College Cork, Cork, T12 YT57 Ireland; 4 IBERS, Aberystwyth University, Aberystwyth, UK; 5 Department of Chemical Sciences, University of Limerick, Limerick, Ireland; 6 Teagasc Food Research Centre, Moorepark, Fermoy, Co., Cork, P61 C996 Ireland; 7 National University of Ireland, Galway, Ireland; 8 Department of Biological Sciences, Munster Technological University, Cork, Ireland; 9 School of Biochemistry, University College Cork, Cork, T12 YT57 Ireland

**Keywords:** Bacteria, Microbial genetics

## Abstract

The human gut microbiome, of which the genus *Bifidobacterium* is a prevalent and abundant member, is thought to sustain and enhance human health. Several surface-exposed structures, including so-called sortase-dependent pili, represent important bifidobacterial gut colonization factors. Here we show that expression of two sortase-dependent pilus clusters of the prototype *Bifidobacterium breve* UCC2003 depends on replication slippage at an intragenic G-tract, equivalents of which are present in various members of the *Bifidobacterium* genus. The nature and extent of this slippage is modulated by the host environment. Involvement of such sortase-dependent pilus clusters in microbe-host interactions, including bacterial attachment to the gut epithelial cells, has been shown previously and is corroborated here for one case. Using a Maximum Depth Sequencing strategy aimed at excluding PCR and sequencing errors introduced by DNA polymerase reagents, specific G-tract sequences in *B. breve* UCC2003 reveal a range of G-tract lengths whose plasticity within the population is functionally utilized. Interestingly, replication slippage is shown to be modulated under in vivo conditions in a murine model. This in vivo modulation causes an enrichment of a G-tract length which appears to allow biosynthesis of these sortase-dependent pili. This work provides the first example of productive replication slippage influenced by in vivo conditions. It highlights the potential for microdiversity generation in “beneficial” gut commensals.

## Introduction

Key factors that determine establishment and maintenance of the different species present within a microbial community in a given host environment largely remain elusive. However, the importance of certain metabolic abilities, such as carbon source utilization, in niche colonization is beyond doubt, thereby creating opportunities for dietary interventions in the case of the gut microbiota^1^. Representatives of the genus *Bifidobacterium* are prevalent and abundant members of the gut microbiota and have been subject to extensive scientific scrutiny, which has revealed various genes relevant to host colonization^2–4^. *Bifidobacterium breve* UCC2003, a nursling stool isolate, has been employed as a human gut commensal prototype to investigate microbe-host interactions relevant to gut colonization^5–7^. Interestingly, certain bifidobacterial species can metabolize (specific) oligosaccharides present in human milk and this ability is believed to at least partly explain their high abundance in breast-fed babies^8,9^.

In addition to resource availability, maintenance of microbiota components in the human gut may be compromised by other factors, such as the sweeping action of the digestive flux in the luminal intestinal tract, which may cause progressive elimination of the microbiota by defecation. In the case of *B. breve* UCC2003, conserved type IVb or tight adherence (Tad) pili have been shown to play an important role in bifidobacterial colonization of the human gastrointestinal tract^6^. In addition to the Tad pili, bifidobacteria may produce so-called sortase-dependent pili^10^. Assembly of a sortase-dependent pilus structure involves expression of a precursor pre-pilin monomer, its subsequent secretion by the Sec pathway, followed by maturation through cleavage at an LPXTG amino-acid sequence by a dedicated sortase to generate a pilus multimer which is covalently linked to the peptidoglycan by a housekeeping sortase [for reviews see^11,12^]. Genomes of members of the *Bifidobacterium* genus may either lack or encompass one or more, genetically distinct, sortase-dependent pilus clusters, of which the corresponding pili are believed to enable specific attachment to various targets of the extracellular matrix, including glycans. They may also be involved in bacterial aggregation^10^. Expression of sortase-dependent pili of *Bifidobacterium longum* appears to be regulated by environmental conditions^13^. In *Lactobacillus rhamnosus* GG, the sortase-dependent SpaCBA pili interact with the C-type lectin DC-SIGN receptor of the host’s dendritic cells, thereby affecting cytokine expression^14^. Sortase-dependent pili in *Bifidobacterium bifidum* not only mediate adherence of this bacterium to proteins of the extracellular matrix or to other bacteria, but also appear to induce a TNF-α immune response^15^.

While bacterial surface-exposed elements may play crucial roles in host interaction and colonization, they may also constitute a target for the host’s immune system to allow host-mediated elimination. Bacteria have developed various mechanisms to evade the host’s immune system. For example, bacteria may ‘hide’ from the host immune system by generating diversity in the expression of certain cell surface-exposed products within their own population. Such diversity is triggered by specific and reversible modification of the genomic sequence involving specialized ON/OFF switch mechanisms, also referred to as phase variation [for a review see^16,17^]. One such mechanism is represented by chromosomal DNA inversion events involving promoter sequences or coding sequences^18^. For example, *Mycoplasma penetrans* modulates multiple surface lipoproteins mediated by a tyrosine recombinase recognizing specific sequences flanking an invertible promoter region^19,20^. Another type of diversity generation is by altering the activity of a given promoter either by epigenetic control or sequence modification. While the former involves methylation of promoter sequences^21,22^, the latter involves specific nucleotide additions or omissions by the DNA polymerase during replication in the spacer region located between the -35 and -10 boxes of a promoter sequence thereby affecting transcriptional efficiency^23^. In *Haemophilus influenza*, such InDel events in a promoter region were found to control the expression of specialized fimbriae genes involved in hemagglutination and adherence activity of *H. influenzae*^24^.This DNA polymerase-mediated InDel generating activity is known as replication slippage, and may also occur at a specific conserved slippage-prone sequence within a coding sequence causing changes in the translational reading frame and derived products. For example, the ruminant pathogen *Mycoplasma agalactiae* exhibits high frequency phase variation involving base addition/omission at a G-tract motif in the coding sequence of *gsmA* which is required for the secretion of the cell-attached β1-6-glucan, a polysaccharide that is targeted by the host immune system^25^. In *Campylobacter jejuni*, a causative agent of human gastroenteritis, phase variation may be an important feature in generating bacterial cell shape diversity^26^, as well as causing surface antigen diversity relevant to escaping the host’s immune response^27^. In *Neisseria* species, O-glycosylation and acetylation of di- and tri- saccharides are both subject to microheterogeneity due to phase variation events occurring at an intragenic G-tract in expression of the corresponding glycosyltransferase and acetylase genes^28^.

In the current work, we investigated an as yet unexplored aspect of micro-diversity generation in members of the genus *Bifidobacterium* involving the bacterial DNA polymerase. DNA polymerase-mediated slippage was studied in the prototype *B. breve* strain UCC2003 at G-tracts found in the sequences encoding two sortase-dependent pilus clusters. In this work, we used Maximum Depth Sequencing (MDS) to discriminate between errors introduced by the host bacterial DNA polymerase from those being introduced by the polymerases employed for DNA amplification and sequencing. Our results indicate that (1) (at least one of the two studied) sortase-dependent pili are involved in gut colonization, (2) expression of the two sortase-dependent pilus clusters appears to be dependent on replication slippage, (3) the slippage event is modulated by the host environment.

## Methods

### Bacterial strains and culture conditions

Bacterial strains used in this study are listed in SI Table S5. *Bifidobacterium breve* UCC2003 (APC microbiome Ireland, strain collection) was routinely cultured in reinforced clostridial medium (RCM; Oxoid Ltd, Basingstoke, Hampshire, United Kingdom) or in de Man Rogosa and Sharpe Medium (MRS) prepared from first principles^29^. Prior to inoculation the MRS was supplemented with cysteine-HCl (0.05% final w/v). Bifidobacterial cultures were incubated at 37 °C under anaerobic conditions using an anaerobic chamber (Davidson and Hardy, Belfast, Ireland).

*Escherichia coli* was cultured in Luria Bertani broth (LB) at 37 °C with agitation. Where appropriate growth media contained tetracycline (Tet; 10 μg/ml), erythromycin (Em; 100 μg/ml for *E. coli*), chloramphenicol (Cm; 5 μg/ml for *E. coli*), or kanamycin (Km; 50 μg/ml for *E. coli*). Recombinant *E. coli* cells containing pORI19 were selected on LB agar containing Em, and supplemented with X-gal (5-bromo-4-chloro-3-indolyl-β-D-galactopyranoside) (40 μg/ml) and 1 mM IPTG (isopropyl-β-D-1-thiogalactopyranoside).

#### Specific conditions for the NGS experiments

A single colony isolate of *B. breve* UCC2003 was obtained from a corresponding bacterial streak on an RCA plate, and then inoculated in RCM and incubated for 14–16 h at 37 °C. Then, the next morning a 2% inoculum was grown in fresh RCM, following late evening by a 1% inoculum in 50 ml MRS supplemented with 0.05% cysteine. The day after (14-16 h later), a 5% inoculum was prepared in mMRS^9^ supplemented with 0.05% cysteine and 1% sugar. A 10% filtered stock solution was prepared for each sugar: glucose, ribose, lactose, maltose, raffinose, and sucrose were purchased from Sigma Aldrich; LNT and LNnT were obtained from Glycom as part of their donation program. For each condition, UCC2003 was cultivated during 6 h.

Animal trial: For monoassociation studies six female, germ free mice (6–8 weeks old), and named M1 to M6, were fed with 1 × 10^9^ of *B. breve* UCC2003 daily on 5 consecutive days. Fecal samples were collected from each mouse at 7 days (time point T1) and 14 days (time point T2) after the last bacterial administration.

### Strain construction

Chromosomal DNA was isolated from bifidobacteria as previously described^30^. Minipreparation of plasmid DNA from *E. coli* was achieved using the Roche high pure Plasmid isolation kit (Roche Diagnostics, Bell Lane, East Sussex, UK any). Procedures for DNA manipulations were performed essentially as described previously^31^. Restriction enzymes and T4 DNA ligase were used according to the supplier’s instructions (Roche Diagnostics). Synthetic single-stranded oligonucleotide primers used in this study were synthesized by Eurofins (Ebersberg, Germany). Standard PCRs were performed using TaqPCR mastermix (Qiagen), while high fidelity PCR was achieved using Q5 polymerase (NEB). *B. breve* colony PCRs were performed according to standard procedures with the addition of an initial single incubation step of 95 °C for 10 min to effect cell lysis. PCR fragments were purified using the Roche High pure PCR purification kit (Roche Diagnostics). Electroporation of plasmid DNA into *E. coli* was performed as described previously^31^. Electrotransformation of *B. breve* UCC2003 was performed as previously described^32^. The correct orientation and integrity of all constructs was verified by DNA sequencing performed at MWG Biotech (Ebersberg, Germany).

Sequence data were obtained from the Artemis-mediated^33^ genome annotations of *B. breve* UCC2003^6^. Database searches were performed using non-redundant sequences accessible at the National Centre for Biotechnology Information internet site (http://www.ncbi.nlm.nih.gov) using Blast. Sequence alignments were performed using the Clustal Method of the MEGALIGN program of the DNASTAR software package (DNASTAR, Madison, WI, USA).

### Construction of *B. breve* UCC2003-113 and UCC2003-1889

DNA fragments encompassing the RBS sequence and 5’ sequences of *bbr_0113* (516 bp) and *bbr_1889* (301 bp) were amplified by PCR using *B. breve* UCC2003 chromosomal DNA as a template and primer pairs 113F and 113R, or 1889F and 1889R respectively (SI Table S4). The polyG sequence in 113F and 1889R was changed to 10 or 9 nucleotides, respectively, so that following homologous recombination, the mutant strains would contain in-frame versions of *bbr_113ab* or *bbr_1889ab* (SI Figure S1). The PCR products generated were ligated to pNZ44, using the unique NcoI and XbaI restriction site that were incorporated into the forward and reverse primers, respectively, and introduced into *E. coli* EC101 by electroporation. Recombinant *E. coli* EC101 derivatives containing pNZ44 constructs were selected on LB agar containing Cm. The expected genetic structure of the recombinant plasmids, pNZ44-113 or pNZ44-1889 was confirmed by restriction mapping and sequencing prior to amplification of the p44 promoter and *bbr_0113* fragment, or the p44 promoter and *bbr_1889* fragment from the pNZ44 constructs with pNZ44F and 113R, or pNZ44F and 1889, respectively. The amplified products were ligated to pORI19, an Ori^+^ RepA^−^ integration plasmid^34^, using the unique HindIII and XbaI restriction sites that were incorporated into the primers and introduced into *E. coli* EC101 by electroporation. Recombinant *E. coli* EC101 derivatives containing pORI19 constructs were selected on LB agar containing Em, and supplemented with X-gal (5-bromo-4-chloro-3-indolyl-β-D-galactopyranoside) (40 μg/ml) and 1 mM IPTG. The expected genetic structure of the recombinant plasmids, pORI19-P44-113 (pORI19 containing p44 promoter and 522 bases of 113), pORI19-P44-1889 (pORI19 containing p44 promoter and 303 bases of 1889) was confirmed by restriction mapping and sequencing prior to subcloning of the Tetracycline resistance antibiotic cassette, tetW, from pAM5^35^ as a SacI fragment into the unique SacI site on each of the pORI19 derivatives. The expected structure of a single representative of each of the resulting plasmids pORI19-P44-113-tet and pORI19-P44-1889-tet (SI Figure S1) was confirmed by restriction analysis. The plasmids were introduced into *E. coli* EC101 harbouring pNZ-M.BbrII-M.BbrIII^7^ by electroporation, and transformants were selected based on Cm and Tet resistance. Methylation of the plasmid complement of such transformants by the M.BbrIII (isoschizomer of PstI) was confirmed by their observed resistance to PstI restriction. Plasmid preparations of methylated pORI19-P44-113-tet or pORI19-P44-1889-tet were introduced by electroporation into *B. breve* UCC2003 with subsequent selection on RCA plates supplemented with Tet. Site-specific recombination in potential Tet-resistant mutant isolates was confirmed by colony PCR using primer combinations tetWFw and tetWRv to verify *tetW* gene integration. The pair of primers “113 confirm” with “p44 confirm”, or “1889 confirm” with “p44 confirm”, were used to verify the correct integration of the recombinant plasmid at *bbr_0113* or *bbr_1889* chromosomal locus, respectively. The resulting PCR products were sequenced to ensure that the recombinant strains harbored the expected in-frame *bbr_113ab* or *bbr_1889ab* genes under the control of the P44 promoter.

### Transmission electron microscopy

The presence of pili on the cell surface of *B. breve* UCC2003-113 or UCC2003-1889 was analyzed by ammonium molybdate staining of bacterial cells and visualized using a Jeol JEM-1400 transmission electron microscope. Briefly cells from an overnight culture were washed once with 0.1M phosphate buffer and fixed 2 h at RT with 2% glutaraldehyde in 0.1M phosphate buffer. After fixation the sample was washed 3 times with 0.1M phosphate buffer and stained with 1% ammonium molybdate for 1 min at RT. The grids were examined, and micrographs visualized, using a JEM-1400 transmission electron microscope (JEOL Ltd., Tokyo, Japan).

### Adhesion assay by viable count method

The adenocarcinogenic cell line HT29 was used to assess the adhesion abilities of *B. breve* UCC2003 and derivative strains. *L. rhamnosus* GG was included as a positive control. HT29 cells were maintained in Dulbecco’s modified Eagle’s medium supplemented with 10% (vol/vol) heat-inactivated (10 min at 70 °C) bovine serum. Adhesion assays were performed essentially as described previously^36^. Briefly, six-well plates were seeded with 1 × 10^6^ HT29 cells per well and were maintained for 12–15 days to allow the cells to fully differentiate. Prior to the assay, the HT29 cell monolayers were washed twice with phosphate-buffered saline (PBS). Overnight cultures of *B. breve* UCC2003, UCC2003-113, UCC2003-1889 or *L. rhamnosus* GG were washed once with PBS, adjusted to an optical density at 600 nm of 1.0, and diluted tenfold in PBS to reach 1 × 10^8^ CFU ml^−1^, as determined by plate counts on reinforced clostridial agar (RCA) for *B. breve* strains, or MRS for *L. rhamnosus* GG. One milliliter of the bacterial suspension was added to the washed monolayers (bacterial cell/epithelial cell ratio of 50:1) and incubated for 1 h at 37 °C (5% CO_2_). Monolayers were washed five times with PBS to remove unbound bacteria. Adherent cells were removed by scraping, serially diluted in PBS, and plated on RCA or MRS as appropriate. The percentage adhesion was calculated as the number of adherent bacteria relative to the number of bacterial cells applied to the HT29 monolayer. Adhesion assays were performed in duplicate in three independent experiments.

### Replication slippage analysis by Maximum Depth Sequencing

(1)* Sample preparation of Maximum Depth Sequencing (MDS) *The MDS protocol is illustrated in Fig. 3 and is based on a previously published methodology^37^. Chromosomal DNA from a bifidobacterial culture grown under in vitro conditions was isolated as previously described^30^, or from a fecal sample using the QiAamp DNA stool kit (Qiagen) as described by the supplier’s instructions. Quantification of DNA was performed with Qubit dsDNA HS Assay Kit (molecular probes life technologies). Digestion of chromosomal DNA strand with an appropriate restriction enzyme (AatII, ZraI, and MluI; SI Table S1) was performed following the supplier’s instruction (Biolabs). MDS was performed on samples derived either from 250 ng of chromosomal DNA (when purified from an in vitro culture sample) or 10 ng when purified from a fecal sample (concentration was 1–2 ng/µl). The digested reaction was purified with High Pure PCR product purification kit (Roche). Incorporation of the 14nt random barcode (“family tag”) involved a “family primer”: its 5’ part sequence contains an NGS adapter P5 sequence following by a 14nt random barcode; its 3’ part sequence is complementary to the 3’ end sequence of the digested chromosomal DNA strand containing the G/C tract sequence of interest (Table S4). Incorporation of the family tag was performed as follow: 22.5 μl of purified digested chromosome was used in a 50 μl reaction volume containing 2.5 μl of 10 μM specific family primer (containing the 14nt random tag) and 25 μl of 2X Q5 High fidelity master mix (Biolabs) to incorporate the family tag at the 3’ end of the digested chromosomal DNA strand of interest. The reaction was incubated successively at 98 °C for 1 min, 60 °C for 15 s, and 72 °C for 1 min. Immediately after this incubation, unused primers were removed by addition of 1 μl Exonuclease and 5.5 μl 10X Exonuclease buffer (Biolabs). Following incubation at 37 °C for 1 h, then 80 °C for 20 min, the product was purified with a PCR clean up kit (Roche) and eluted in 27 μl H20. Linear amplification using forward adapter amplifier primers: The purified product was then used as a template for linear amplification. Reaction contains 22.5 μl of purified template, 2.5 μl of 10 μM primer F286, and 25 μl of 2X Q5 High fidelity master mix (Biolabs). Incubation was 13 cycles of: 98 °C for 5 s, 61 °C for sec, and 72 °C for 15 s. Exponential amplification: To the linear amplification reaction, 2.5 μl of 10 μM of each primer (a gene specific reverse primer (SI Table S4) and the reverse NGS adapter primer R287) was added. Incubation was performed for 16 cycles: 98 °C for 15 s, 61 °C for 15 s, and 72 °C for sec. The expected product was then purified on a 1% TAE agarose gel using the High Pure PCR clean up kit (Roche). Elution was with 15 µl or 30 µl H20 depending on the relative quantity of the expected product detected on a 1% agarose gel stained with Safeview (NBS Biologicals ltd manufacter, NBS-SV1).

Subsequently, a limited cycle PCR using Nextera XT indices (Illumina) was performed to incorporate sequencing adaptors and dual-index barcodes to each PCR product. Products were then purified with Ampure XP beads (Beckman Coulter), quantified using the Qubit fluorimeter (Thermo Fisher) and pooled at an equimolar concentration. The mix of samples was sequenced on a MiSEq (Illumina) mid input (300 cycles) with V2 chemistry (Illumina).

(2) *Bioinformatic analysis of NGS sequences derived from Maximum Depth Sequencing *A first set of NGS sequencing involved 40 samples analyzed on an Illumina platform using 150 bp pair-ended sequencing (Teagasc sequencing platform, Ireland). This set included samples 1–36, 41–42, 47–48, each having between 2 and 4 million reads. A second set of NGS sequencing involved 30 samples analyzed on an Illumina platform using 150 bp paired-end sequencing. That second set included samples 37–40, 43–46, 49–54, and 95–100, and yielded approximately 1 million reads per sample. For each sample, the following are indicated in SI Table S1: the name of the sample with corresponding source of UCC2003 chromosomal DNA including bacterial cultivation condition, the chromosomal DNA strand analyzed by Maximum Depth Sequencing, the number of NGS reads, the number of sequences for each MDS family and sub-family.

Analysis of Indel in G-tract: First, the family tag (14nt random sequence) was located and extracted by performing a Blastn alignment for both NGS reads R1 and R2 independently. Then, NGS reads containing “G’s end” were removed. The remaining NGS reads were subsequently filtered to retrieve those containing a homopolymeric G/C-tract from those where the G/C-tract contained one or more than one base G substituted by a base C, G or T. The identity of the G-tract was also retrieved with the corresponding family tag, and the NGS reads were clustered with the family tag.

For the specific analysis of InDel by MDS aims to discriminate between the error derived from the bacterial host polymerase from those introduced by in vitro PCR reactions, clusters were selected when containing at least 3 NGS reads. These family sequences were subsequently filtered when a representative sequence was selected. Such a representative sequence corresponds to the identical sequence whose statistical value of mode and median were equal. Finally, family sequences were clustered together when they shared an identical G-tract. The number of family sequences (each reflecting a unique chromosomal molecule) associated with a specific G-tract length is indicated in SI Table S1.

### Bioinformatic search of G-tract sequences in *Bifidobacterium* strains

Fully sequenced and publicly available *Bifidobacterium* genomes (Table 1) were searched for the presence of sortase-dependent pilus clusters, particularly a sortase-encoding gene (PF04203) with associated surface proteins. The identified clusters were searched for the presence of a G-tract sequence up to 500 bp upstream and 200 bp downstream the predicted gene start in the first gene of the operon.

**Table 1:** G-tract mapping in sortase dependant pilus genes specified in other *Bifidobacterium* strains.

Species	Strain	Sortase pilus clusters (total)	PolyG pilus locus	PolyG length (bp)	PolyG position	Strand
*Bifidobacterium longum*	NCIMB8809	1	B8809_1831-29	12	2303726–2303737	−
	NCC2705	1	BL0674-76	11	1662690–1662700	+
	DJO10A	1	BLD_1467-69	10	1704297–1704306	+
	BBMN68	1	BBMN68_1404-06	10	1679020–1679029	+
*Bifidobacterium breve*	UCC2003	2	Bbr_0113-15	11	164606–164616	+
			Bbr_1889-07	10	2376297–2376306	−
	JCM 7017	2	B7017_0130-32	11	170185–170195	+
			B7017_2091-93	10	2242600–2242609	−
	NCFB 2258	2	B2258_0100-02	11	133056–133066	+
			B2258_1894-96	10	2272238–2272247	−
	215W447a	2	BB215W447A_0128-30	10	164763–164772	+
	082W48	1	BB082W48_0128-30	9	164929–164937	+
	NRBB02	1	NRBB02_0129-31	9	164595–164603	+
*Bifidobacterium dentium*	NCTC11816	5	NCTC11816_00529-31	10	629858–629867	+
			NCTC11816_01833-35	13	2144867–2144879	−
	Bd1	7	BDP_0534-38	11	629857–629867	+
			BDP_1874-76	13	2145404–2145416	−
	E7	5	J7M36_02635-50	10	633200–633209	+
	N8	6	J7M35_10275-90	11	2468221–2468231	−
*Bifidobacterium pseudocatenulatum*	DSM 20438	2	BBPC_1756-59	11	2194167–2194177	−
	YIT11956	5	KYE72_04985-10	11	1186707–1186717	+
			KYE72_09605-25	10	2289128–2289137	−
	N8	6	J7M35_08440-50	10	2034919–2034928	−
			J7M37_09250-70	11	2242771–2242781	−
*Bifidobacterium bifidum*	PRL2010	3	BBPR_1820-22	9	2185007–2185015	−
	HN002	3	JQN90_03530-50	8	868939–868946	−
	BGN4	3	BBB_1816-20	10	2194102–2194111	−

Columns from left to right indicate the name of the bacterial species and strains, the number and name of sortase-dependent pilus cluster(s) containing a G-tract of specific nt length with the corresponding genomic coordinates and chromosomal DNA strand.

### Ethics approval and consent to participate

All experiments using mice were approved by the University College Cork animal ethics committee and all methods were performed in accordance with the relevant guidelines and regulations. The study is reported in accordance with ARRIVE guidelines.

## Results

### Replication slippage allows functional assembly of sortase-dependent pilus

**Figure 1:**
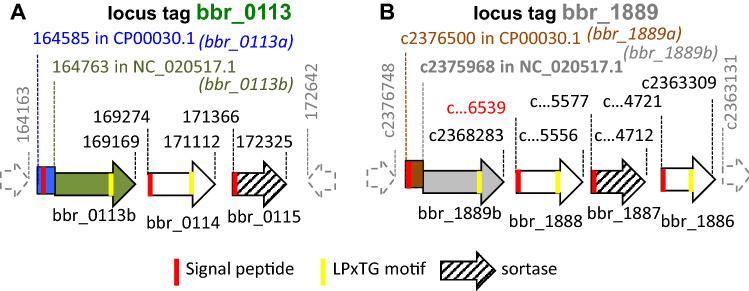
Sortase-dependent pilus gene clusters of UCC2003. Genetic organization of the locus containing *bbr_0113* (**A**) and *bbr_01889* (**B**). Coding sequences are indicated with an arrow, with the annotated *bbr_0113* (green) *bbr_1889* ORF (grey) re-named *bbr_0113b* and *bbr_1889b*. The corresponding upstream ORF is named *bbr_0113a* (blue) and *bbr_1889* (brown). Other sortase-dependent pilus genes are highlighted in white and the dedicated sortase enzyme by a stripped arrow. Signal peptide and LPTxG motifs are indicated by vertical red and yellow bars respectively. The UCC2003 genomic coordinates of NC_020517.1 are indicated above the arrow.

The *B. breve* UCC2003 genome contains two loci, corresponding to locus tags *bbr0113*-*bbr0115* and *bbr1889*-*bbr1886*, each of which is predicted to encode the biosynthetic machinery for sortase-dependent pili. They consist of a cluster of three and four genes, respectively. These two gene clusters contain a basic arsenal of functional elements allowing gene expression, protein secretion and polymerization required for pilus assembly (Fig. 1, panels A&B). It includes genes encoding: (1) a dedicated sortase, a key enzyme involved in polymerization of pilin protein sub-units by a transpeptidation reaction at an LPXTG motif^38,39^, (2) the LPXTG motif present in each of the pilin subunits, (3) the presence of a Sec secretion signal specified by each gene, being required for export to the bacterial cell surface.

Interestingly, the first product of each cluster, a pilin subunit precursor, has the Sec secretion signal and LPXTG motif encoded by two distinct ORFs separated by a sequence containing a homopolymeric G-tract. A potential re-framing mechanism at this G-tract to join the two ORFs into a single CDS was considered in the original annotated version of GenBank submission CP000303.1 (updated 31st January 2014) of the UCC2003 genome, though was not considered in the most recent update NC_020517.1 (on 30th March 2017). Here, the former *B*. *breve* UCC2003 ORFs containing the coding sequences [coordinates 164585-164608] and [coordinates 164607-169169] are re-designated as ORFs *bbr_0113a* (the ‘upstream ORF’) and *bbr_0113b* (the ex-*bbr_0113*), respectively; and those representing the sequences [2376304-2376500] and [2368283–2376305] are re-named as *bbr_1889a* (the ‘upstream 65-AA encoding ORF’) and *bbr_1889b* (the former 2674-AA encoding *bbr_1889*)*.*

The *bbr_0113a* and *bbr_1889a* genetic elements are highly transcribed, and transcription is strongly reduced downstream of the 3’ stop codon in both *bbr_0113a* and *bbr_1889a*^39^. Interestingly, a ‘dispersed’ profile of transcription termination sites can be observed within the 600nt sequence downstream of the stop codon of *bbr_0113a* or *bbr_1889a*^40^. This is indicative of a progressive release of the RNA polymerase from the DNA template by a polarity effect rather than by the presence of a specific transcriptional termination signal which would have been characterized by a much more defined termination site. While in NC_020517.1 *bbr_0113b* (ex-*bbr_0113*) and *bbr_1889b* (ex-*bbr_1889*) have annotated ‘start codons’, the coding sequence surrounding these ‘start codons’ lack a canonical Shine Dalgarno sequence and predicted (N-terminal) Sec secretion signal. Therefore, the apparent lack of translational signals to allow pilus expression, and the observed polarity effect provides evidence for lack of translational initiation at those sites.

**Figure 2:**
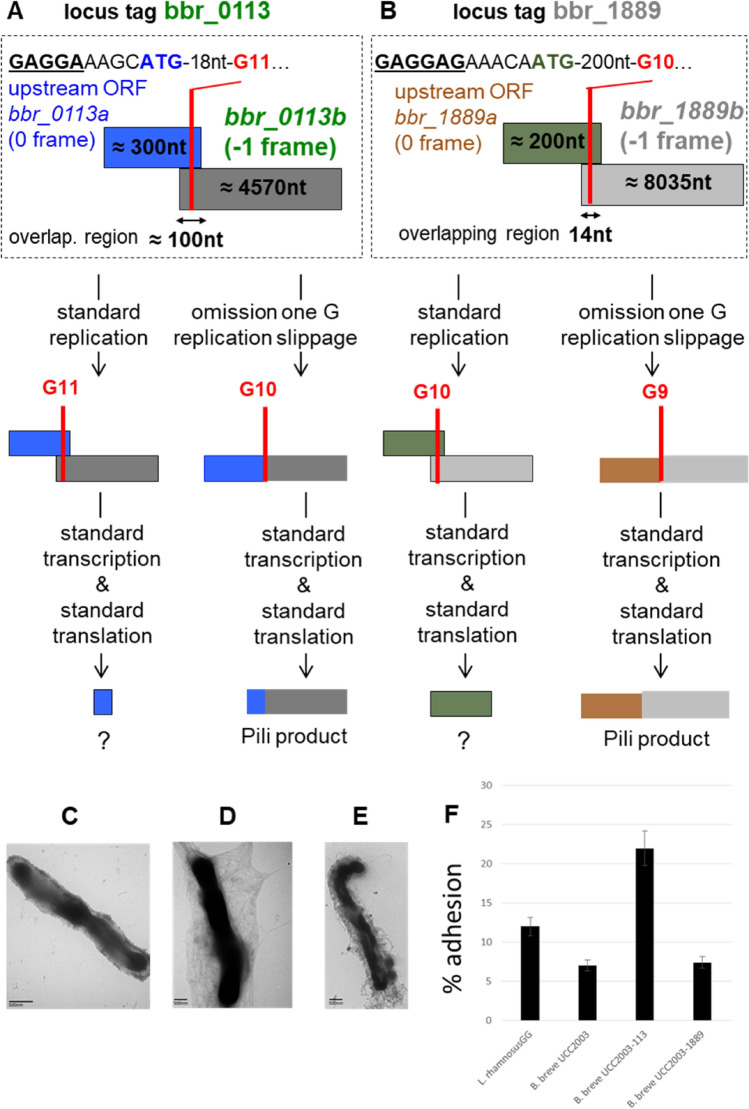
Phenotype and biological activity. A schematic of the expression of Bbr_0113ab and Bbr_1889ab pilus products derived by replication slippage is indicated in (**A**) and (**B**) where ORFs are indicated by a rectangle, for *bbr_0113* locus (**A**) with *bbr_0113a* (in blue) and *bbr_0113b* (in green), for *bbr_1889* locus (**B**) with *bbr_1889a* (in brown) and *bbr_1889b* (in grey). Above is indicated a presumably initiation of translation encompassing the translational codon for *bbr_0113a* and *bbr_1889b* (indicated in same color as their ORF) and a potential Shine-Dalgarno sequence (underlined). Below is shown the impact of standard or adequate replication slippage causing, respectively, the expression of a truncated or complete pilus product. Expression of sortase-dependent pili by recombinant strains of (**C**) *B. breve* UCC2003, (**D**) UCC2003-113 or (**E**) UCC2003-1889. Cells were stained with ammonium molybdate and visualized by transmission electron microscopy. Biological functionality of pili in cell interaction of (**F**) *B. breve* UCC2003, UCC2003-113, UCC2003-1889 to HT29 cells as determined by the viable count method. Data presented are an average of triplicate experiments. *L. rhamnosus* GG was included as a control.

Since a (strong) active promoter^40^ and translation initiation sequences are available for expression of *bbr_0113a* and *bbr_1889a*, we first explored if appropriate omission or addition of base(s) would lead to the successful assembly of surface-exposed pili. Pilus production was examined in derivatives of *B. breve* UCC2003 in which *bbr_0113a* and *bbr_0113b*, or *bbr_01189a* and *bbr_1889b*, had been placed in-frame by insertional mutagenesis at their corresponding chromosomal loci (SI Figure S1 & Materials and Methods). The presence of pili was analyzed by ammonium molybdate staining and visualized by transmission electron microscopy. In contrast to the parent strain, *B. breve* UCC2003, apparent pilus-like structures were observed on the cell surface of UCC2003-113 or UCC2003-1889 (Fig. 2, panels C-E). As expected such alteration of the number of Gs allows expression of the fused genes. It apparently also results in expression of the 3’-located genes of the two pilus gene clusters, including the corresponding dedicated sortase-encoding gene, thereby apparently allowing the production of surface-exposed pilus structures.

### Adhesion of *B. breve* strains to HT29 epithelial cells

We next explored the biological properties of each of the two sortase-dependent pili to mediate cell surface adhesion. Strains *B. breve* UCC2003 (WT), *B. breve* UCC2003-113 and *B. breve* UCC2003-1889 were tested for their ability to adhere to HT29 epithelial cells. *B. breve* UCC2003 and UCC2003-1889 exhibit similar adhesion abilities with approximately 7% of bacterial cells adhering to the HT29 cells, while the adhesion ability of UCC2003-113 was significantly higher, with more than 20% of bacterial cells adhering to HT29 epithelial cells (Fig. 2, panel F). This data indicates that expression of sortase-dependent pili, at least by *B. breve* UCC2003-113, promotes significantly enhanced adhesion to epithelial cells. This apparent pilus-mediated adhesion is likely to be an advantageous feature for bifidobacteria in the context of host colonization and host–microbe interaction.

### Analysis of replication slippage by Maximum Depth Sequencing

Considering that the length of a homopolymeric G/C tract is presumed to determine its propensity for DNA polymerase-mediated slippage, a Maximum Depth Sequencing (MDS) approach was performed^37^ to allow discrimination between errors introduced by the DNA polymerases required for in vitro amplification and sequencing from those generated by the bifidobacterial host DNA polymerase during genome replication.

**Figure 3:**
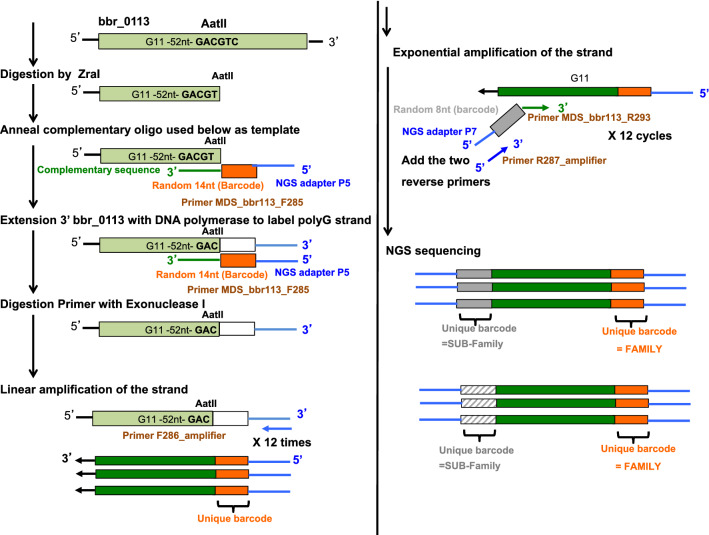
Maximum Depth Sequencing (MDS). The chromosomal DNA strand containing the sequence of interest, such as G11 of bbr_0113, is digested 3’ by a restriction enzyme (AatII). A synthetic oligonucleotide containing 5’ the NGS adapter P5 sequence (blue line), 14nt random sequence “family tag” (orange rectangle) and a sequence (green line) being reverse and complementary to the sequence of the newly produced 3’end of the chromosome strand, is used as template to incorporate the family tag and adapter sequence 3’ to the sequence of interest. Un-incorporated oligos are digested by Exonuclease I. Then, 12 cycles of linear amplification are performed with a specific “primer amplifier” complementary to the newly neo-synthesized DNA 3’end. Corresponding products are then used as template in exponential amplification involving the addition of a second primer. It contains 5’ the NGS adapter P7 sequence (in blue), an 8nt random sequence but can be facultative (in grey rectangle), and a sequence (in green) being reverse and complementary to a specific sequence of the sequence of interest. The purified PCR product is then subjected to NGS sequencing with the family tag reflecting a unique chromosomal DNA strand molecule.

MDS focuses on the analysis of a sequence of interest (in our work a G- or C-tract) present in one chromosomal DNA strand (Fig. 3), and is based on the incorporation of a random tag (named “family” tag) 3’ to the region of interest prior to any amplification. This incorporation involves the generation of a new 3’ DNA end which is specifically extended by polymerase synthesis to incorporate the family tag specified by a DNA oligonucleotide template (Fig. 3). Next, each chromosomal sequence encompassing the region of interest associated with its unique tag is used as a template in a linear amplification reaction to generate a pool of single-stranded sequences. This maximizes recovery of the same sequences associated with a unique family tag. Then, the pool is used as template for an exponential amplification step to generate a double-stranded product suited to Next Generation Sequencing. The derived amplicon products of ≈150 bp encompassing the nucleotide tract and the family tag, were subjected to a 150 bp paired-end sequencing on an Illumina platform in order to maximize the recovery of the “family” tag and the associated G or C tract (see Materials and Methods). The determination of the identity of the original chromosomal sequence of interest (G or C tract) is now possible by retrieving from each family, a corresponding sequence whose occurrence within a particular family is statistically representative of the region of interest in the original chromosomal molecule^37^.

In the current work, the identity of the tract (being G’s or C’s) is indicated with respect to the chromosomal DNA strand used to perform MDS analysis. Therefore, the restriction site indicated in MDS experiments is located 3’ to that tract (on the original chromosomal locus) and was used to incorporate a 14nt random tag (‘Family tag’) and the P5 NGS adapter containing the sequence of the NGS primer R1 (generating “R1” reads during paired-end Illumina sequencing). Therefore, the Illumina sequencing reaction performed with primer R1 uses a DNA template containing the same identity of base sequence as the chromosomal DNA strand analyzed by MDS. The corresponding NGS reads generated with primer R1 have the general sequence: (from 5′ to 3′) the 14nt tag (Family tag), followed by the G/C tract (the sequence of which represents the complementary and reverse sequence of the tract present in the original chromosomal strand used to perform MDS).

**Figure 4:**
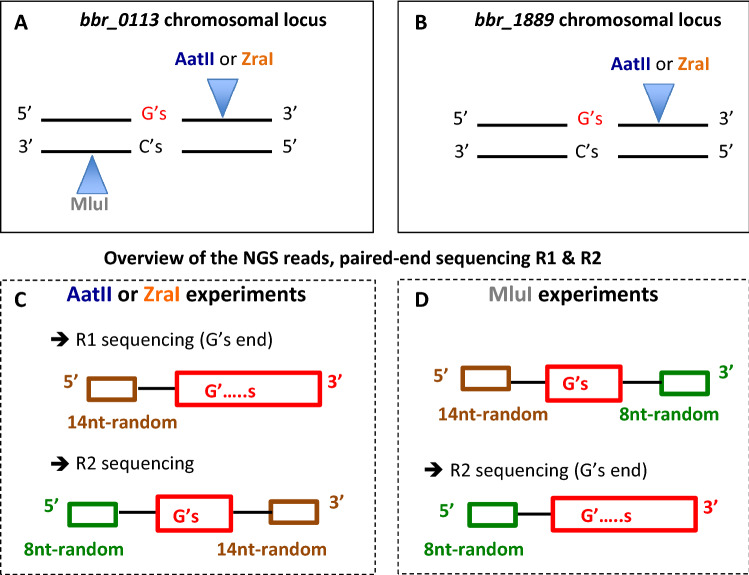
G’s end phenomenon. The position of available restriction sites present in the double stranded chromosomal locus of *bbr_0113* (**A**) and bbr_1889 (**B**) used to generate a newly 3’end, downstream the sequence of interest, of one of the two chromosomal DNA strands are indicated. Because this 3’ end incorporates a 14nt random tag and the complementary sequence of the NGS primer R1 during the first step of MDS, the corresponding NGS reads generated using R1 will sequence first the 14nt family tag, followed by the tract, and then the 8nt tag. When MDS targets a chromosomal strand containing a G-tract (using either AatII or ZraI), the NGS read generated with primer R1 causes a G’s end phenomenon, which does not occur for primer R2 (**C**). Conversely, when MDS focus on a chromosomal DNA strand containing a C-tract (MluI), the G’s end phenomenon occurs with R2 but not with R1 (**D**).

**Figure 5:**
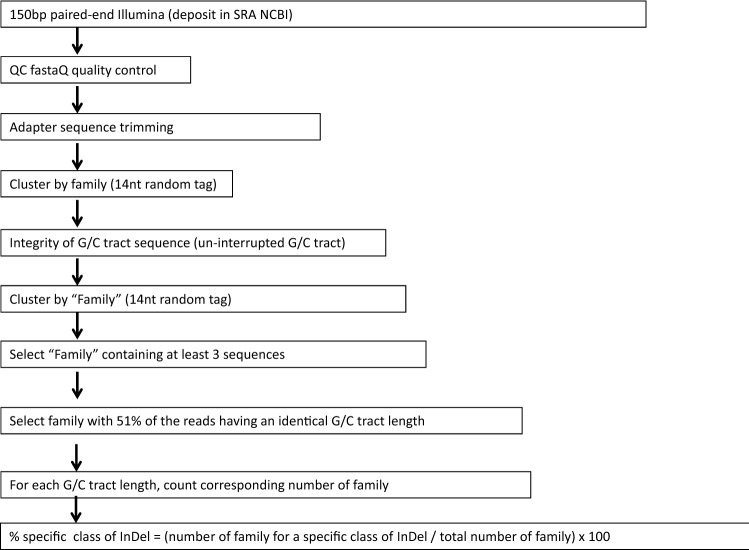
MDS bioinformatic workflow. Selection and clustering of the “family” sequences containing a specific G/C tract specified at *bbr_0113* and *bbr_1889* chromosomal loci.

A 150 bp paired-end Illumina sequencing was performed to exclude sequencing errors within the G- or C-tract specified by either of the two DNA strands of the PCR product. Unexpectedly with the G-tract containing template, the counterpart sequence of the derived NGS reads through and beyond the G-tract template sequence, corresponds to repetitive G base(s), named in this current work “G’s end (Fig. 4). This phenomenon is believed to be caused during Illumina sequencing of a G-tract-containing template due to either a G-tract-forming secondary structure thereby forming a physical roadblock for the DNA polymerase or to non-specific, repetitive binding of fluorescent Guanine nucleotide. Therefore, the bioinformatic analysis of the paired-end NGS data was performed with only one of the two NGS reads, R1 or R2. The general workflow of the MDS bioinformatic analysis is shown in Fig. 5. The description of the NGS samples can be found in SI Table S1, as well as details of the bioinformatics corresponding to the occurrence of the “G’s end” phenomenon (SI Table S2), and the generation of the “family” reflecting a sequence of interest derived from one specific chromosomal molecule (SI Table S3).

### MDS analysis of InDel at the *bbr_0113* and *bbr_1889* chromosomal loci

To validate the MDS method with the analysis of the identity of the G/C tract at the *bbr_1889a-1889b* locus, two control experiments were performed. Each control included a chemically synthesized oligonucleotide, PAGE purified, containing the *bbr_0113* locus sequence specified by one of the chromosomal DNA strands. One of the oligonucleotides has a G11 sequence (SI Table S4, oligo F449) and the other has a C11 (oligo F450) tract. Each oligonucleotide was used as a template and tested using a 100-fold range of two concentrations (see Materials and Methods). The NGS paired-end sequencing of the derived PCR product reveals the presence of the “G’s end” phenomenon when the DNA strand (of the PCR product) used as a template contained a G-tract. Therefore, the reads exhibiting such “G’s end” were discarded, while the remaining reads were analyzed.

**Figure 6:**
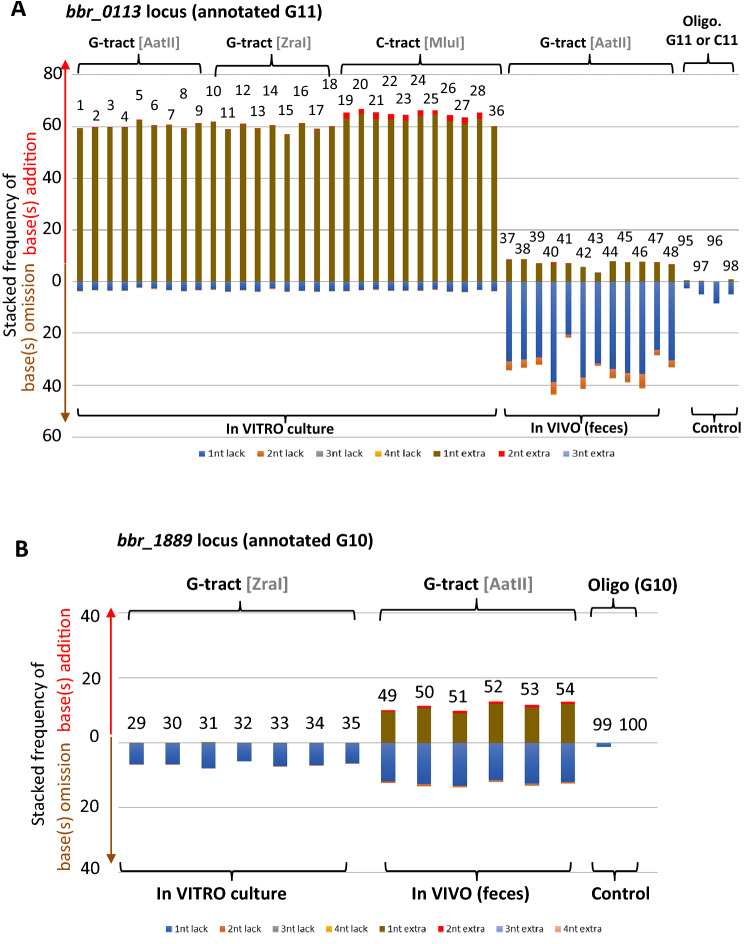
Replication slippage analysis by Maximum Depth Sequencing (MDS). The stacked frequency of inDel at G/C tract of *bbr_0113* locus (**A**) and *bbr_1889* locus (**B**) is indicated for each specific number of base(s) addition or omission, with the frequency of the annotated nt length being omitted. The MDS analysis reveals the distribution of InDel derived from only one of the chromosomal DNA strands specifying the G-tract (DNA coding strand) or the C-tract (non-coding DNA strand). The identity of the chromosomal DNA strand with the restriction site used for the MDS is indicated at the top. The general cultivation condition of UCC2003 for each sample (numbering above each stacked frequency bar) is shown at the bottom, with the specific cultivation conditions also indicated in SI Table S1. The condition ‘control’ refers to a control experiment involving chemically synthesized oligonucleotides specifying the region of interest encompassing either the forward or reverse chromosomal DNA strand of *bbr_0113* or *bbr_1889* locus.

The NGS results for the G-tract-containing synthetic template correspond to samples 95 & 96, and for the C-tract-containing synthetic template to samples 97 & 98 (Fig. 6A). Within each set, the first sample represents sequencing data when a 100-fold higher concentration of oligonucleotide template was employed compared to the second sample. With the G11-tract control, sequencing reads from sample 96 showed the presence of just 36 MDS families (after bioinformatic filtering). Sample 95 was shown to correspond to 808 MDS families exhibiting a frequency of base addition of ≈0.5% and a base omission frequency of ≈2.7%. For the C11-tract control, the corresponding number of NGS families for samples 97 and 98 were shown to be 240 and 528, respectively. The result shows a similar frequency of slippage distribution with ≈94% having the expected number of G’s within the G-tract, ≈5% having one base omission (G10), and 1% having one base addition (G12), (Fig. 6A). Because the MDS allows discrimination between errors due to the polymerase reagent, this implies that ≈3.2% of the oligonucleotide F449 is heterogeneous at the G-tract, and ≈6% of oligonucleotide F450 is heterogeneous at the C-tract. This is presumed to be due to errors in chemical synthesis of an oligonucleotide containing a homopolymeric sequence. Chemical synthesis of oligonucleotides involves nucleotide cycle addition at the 3’ end of the nucleic acid and the incorporation of the cognate nt at each cycle is not 100% efficient at each cycle^41,42^. Therefore, it should be expected that without purification the oligonucleotide population contains a mix of sequences having at least one base less at the homopolymeric sequence. Though the oligonucleotide encompassing the expected sequence was PAGE purified, it cannot be excluded that potential secondary structure formation of the oligonucleotide may have affected its migration within the PAGE, generating a PAGE-purified product that contains a mixed population of varying homopolymeric nt length.

Then, the MDS method was executed employing chromosomal DNA from *B. breve* UCC2003 cultivated in complex MRS media. A single colony grown on an RCA plate was used to prepare a subculture prior to inoculation in MRS liquid culture (see Materials and Methods). Chromosomal DNA was then purified from the bacterial culture when in exponential phase of growth to perform the MDS analysis. In the case of the *bbr_0113a-0113b* locus, the NGS result of the chromosomal DNA strand containing the G-tract sequence, involving MDS performed with the AatII site (Fig. 3), was considered only with R2 reads. The obtained results show that 37% of the population contains a G11 tract which is the number of annotated G’s, 59% has a G12 tract (an extra G), and 3% possesses a G10 sequence (one base omission) (Fig. 6A, sample 1). Confirming the above result is the counterpart MDS experiment performed on the complementary chromosomal DNA strand containing the C-tract using MluI. Analysis of the NGS reads R1 revealed a slippage distribution at the C-tract that was consistent with that found at the G-tract (Fig. 6A, sample 19).

To summarize, the consistency of the *bbr_0113* MDS results obtained (1) from the PCR products derived from the sequence present in a given chromosomal DNA strand, and (2) from utilization of one of the NGS reads of a paired end sequencing set (R1 or R2), demonstrates that the MDS method is a robust approach to determine the length of a G/C tract at a specific location in a particular chromosomal strand. Therefore, the sum (63%) of the stacked frequency of base(s) additions and omissions (Fig. 6A) implies that 37% of the assessed *B. breve* UCC2003 population possesses a *bbr_0113a-0113b* locus with its number of G’s identical to the number of G’s annotated in the Refseq sequence (NC_020517.1).

With the *bbr_1889a-1889b* locus, a control experiment for the MDS method for the *bbr_1889* was included. It involved a chemically synthesized, PAGE purified oligonucleotide template containing a sequence corresponding to the *bbr_1889* section with the G10-tract (SI Table S4, oligo F451). The corresponding sample names are 99 (corresponding to 156 MDS families passing the bioinformatic filter) and 100 (with just 19 identified MDS families). Similar to the situation for *bbr_0113*, the NGS paired-end sequencing exhibits “G’s end” with R1 reads (SI Table S2). Therefore, only the NGS reads generated by R2 were considered for the analysis. With sample 99 (tested with 100-fold higher concentration of a synthetic template), the result showed that ≈99% of single family-associated reads possess the G10 tract (the expected number of G’s), while just 1% contain a G9 sequence configuration (Fig. 6B, sample 99).

Analysis at the *bbr_1889a-1889b* locus of the chromosomal DNA strand containing the G-tract involved the restriction site ZraI. The results show that ≈93.4% contains G10 the number of G’s annotated for the *bbr_1889a-1889b* locus, and ≈6.5% have G9, i.e. corresponding to a single base omission (Fig. 6B, sample 29). Base addition was determined to be ≈0.1%, therefore representing a small minority of the assessed population. This indicates that most cells of the analyzed UCC2003 population contain a *bbr_1889a-1889b* locus with a G-track of 10 Gs, which is identical to that annotated in the Refseq sequence (NC_020517.1).

In conclusion, the MDS results indicate that the UCC2003 population under the conditions tested exhibits a strong heterogeneity in the number of G’s at the *bbr_0113a-bbr_0113b* locus but much less so at the *bbr_1889a-1889b* locus. This indicates that G11, at least within the *bbr_0113a-0113b* genetic context, represents a strong slippage motif when compared to a G10 motif. This slippage heterogeneity which exhibits a higher proportion of G12 tract (60%) within the population rather than the annotated G11 (37%) points to high frequency slippage at G11 of the *bbr_0113a-0113b* locus in our experimental condition or a limitation of the sequencing/assembly method when the *B. breve* UCC2003 genome sequence was originally determined (NC_020517.1).

### Replication slippage frequency with different carbon sources

Considering the possibility of potential stimulation of slippage by a nutrient present in the MRS medium (a rich and complex growth medium containing both yeast extract and glucose) due to the surprisingly heterogeneous variation in the length of the G-tract found at the *bbr_0113* locus, we explored the impact of a more defined medium on the frequency of replication slippage. Because *B. breve* UCC2003 is known to be able to utilize a large variety of sugars, including certain Human Milk Oligosaccharides (HMOs;^9^, we tested minimal MRS (absence of yeast extract) supplemented with a specific sugar as the sole carbon source.

We assessed the G-tract in the *bbr_0113a-0113b* locus by MDS analysis following growth of *B. breve* UCC2003 on the following carbohydrates: lactose (Fig. 6A, samples 3 &12 for the chromosomal strand containing the G-tract, vs sample 22 for the other strand containing the C-tract), glucose (samples 4 &13, vs 23), ribose (samples 11, vs 21), sucrose (samples 5 &14, vs 24), maltose (samples 8 &17, vs 27), raffinose (samples 9 &18, vs 28), and the HMOs lacto-N-tetraose (LNT; samples 6 &15, vs 25), and Lacto-N-neotetraose (LNnT; 7 &16, vs 26). Interestingly, all analyzed samples were shown to exhibit a very similar distribution of frequencies of base(s) additions and omissions when compared to those found in glucose-containing media (see samples 1 &19, as discussed above). Notably, independent of the carbohydrate used for growth of *B. breve*, we observed a slightly more frequent addition of one C base, and more frequent addition of two C bases within the chromosomal DNA strand containing the C-tract when compared to the strand containing the G-tract (see “Discussion”).

Next, we analyzed the G-tract in the *bbr_1889a-1889b* locus for *B. breve* UCC2003 cultivated on the following carbohydrate: lactose, sucrose, LNT, LnNT and raffinose (Fig. 6B, samples 31, 32, 33, 34 & 35, respectively). All assessed samples exhibit similar results as those found in glucose-containing medium (previous sample 29).

In conclusion, the replication slippage at the *bbr_0113* or *bbr_1889* loci does not appear to be modulated by any of the carbon sources tested. Importantly, the results obtained under these varying medium conditions show a consistent discrepancy between the identity of the G-tract at *bbr_0113* locus being annotated as G11 in the Refseq sequence NC_020517.1, and the two major G-tract populations, i.e. G11 (present at ≈37%) and G12 (present at ≈60%), encountered in the assessed UCC2003 cell cultures (see “Discussion”).

### The gastrointestinal environment stimulates replication slippage at G-tracts present in two sortase-dependent pilus loci

Assuming that bifidobacterial sortase-dependent pili, which represent surface-exposed appendages, convey a biological attachment property, it may be that pilus expression is stimulated within the gastrointestinal tract of the mammalian host. Therefore, replication slippage was investigated in a murine model.

**Figure 7:**
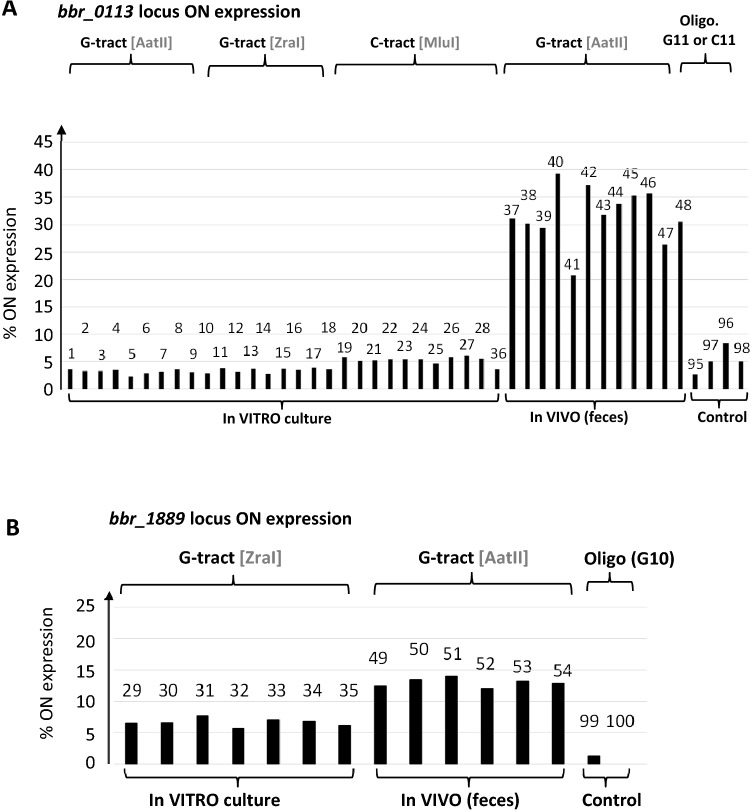
Replication slippage-mediated “ON” expression. The addition of (−1+3n) frequency of indel at G/C tract of *bbr_0113* locus (**A**) and *bbr_1889* locus (**B**) and multiplied by 100 to yield the percentage of ON expression. The percentage of ON/OFF ratio for *bbr_0113* locus (A) and *bbr_1889* locus (B) was calculated as follow: (number tracts giving ON expression /total number all tracts) * 100.

We performed MDS analysis of the chromosomal DNA strand specifying the G-tract of the *bbr_0113a-0113b* locus, thus involving NGS reads derived from primer R2 of the NGS paired-end sequencing. The chromosomal DNA was purified from fecal samples of six mice (named from M1 to M6). Each was analyzed at 7 days (named T1) and 14 days (named T2) following administration of *B. breve UCC2003*: M1 (sample 37 at T1 vs sample 43 at T2), M2 (38 at T1 vs 44 at T2), M3 (39 vs 45), M4 (40 vs 46), M5 (41 vs 47) and M6 (42 vs 48). The MDS results reveal a similar slippage pattern in all six mice (Fig. 6A). The frequency of base(s) omission is at least 4 times higher than that of base(s) addition. Omission of one base (≈30%) is approximately 10 times more frequent than omission of two bases (≈3%). Interestingly, comparison of time points T1 and T2, show that for mice M2, M3, M5 & M6, the frequency of base(s) omission tends to slightly increase with their corresponding overall frequency of base addition remaining similar. For mice M1 and M4, the overall frequency of base omission slightly decreases between time point T1 and T2, with for mouse M1 a decrease in the frequency of base addition. To summarize, while some variation is observed between individual mice, the results show a strong and consistent slippage pattern of Indel base(s) distribution with base omission(s) being more preponderant than base addition(s). It also indicates that within each mouse, 30% of the UCC2003 population contains a single base omission resulting in a G10 tract at the *bbr_0113* locus. With the genotype G10, *bbr_0113a* and *bbr_0113b* fuse to form a single ORF (Fig. 1A) whose translation would lead to the production of sortase-dependent pili (Fig. 7). This genotype is therefore 10-times more preponderant in the gastrointestinal environment compared to when assessed under in vitro growth conditions.

To assess the *bbr_1889a-1889b* locus under in vivo conditions, MDS was performed on chromosomal DNA purified from the fecal samples of four mice with time points for the following samples: M1 (sample 49 at T1), M2 (sample 50 at T1 and sample 52 at T2), M3 (sample 53 at T2) and M4 (sample 51 at T1 vs sample 54 at T1). The obtained results show a very similar slippage pattern among the samples from different mice. Interestingly, the slippage occurs in both directions with the overall frequency of base addition(s) and omission(s) being similar ≈10–12% (Fig. 6B). This in vivo result contrasts with that found for the in vitro culture where slippage was shown to mostly generate base omission(s) rather than base addition(s). With a single base omission, the nt length of the G-tract (G9) tract allows fusion of *bbr_1889a* and *bbr_1889b* into a single ORF (Fig. 1B) whose translation is expected to facilitate expression of the corresponding sortase-dependent pili. Interestingly, the occurrence of this G9 tract is about two times more frequent under in vivo conditions compared to the in vitro cultivation condition.

In conclusion, replication slippage events (of −1+3n; with n being an integer) that lead to ORF fusion i.e. generation of uninterrupted Bbr_0113ab, or Bbr_1889ab, appear to be enriched under in vivo (gastrointestinal) conditions when compared to the in vitro cultivation conditions. This enrichment is twofold for *bbr_1889ab* and tenfold for *bbr_0113ab* (Fig. 7).

### G-tracts are found in other sortase-depending pili genes in *Bifidobacterium* strains

The presence of homopolymeric sequences was explored through a computational search in other sequenced *Bifidobacterium* strains (Method). The result (Table 1), revealed that this apparent pilus-associated, replication-mediated frameshift mechanism is observed across various representatives of *Bifidobacterium* species. *B. breve* and *B. dentium* were the species harbouring either one or two clusters with associated predicted poly-G stretches. *B. longum*, *B. bifidum* and *B. pseudocatenulatum* showed limited occurrence of G-tracts in pilus biosynthesis gene clusters, generally restricted to a single cluster per genome. The obtained results suggest that while not all pilus-specifying gene clusters contain G-tracts, this genetic feature is indeed commonly observed in a number of clusters across various members of this genus.

## Discussion

Productive utilization of a (programmed) slippage-prone motif, especially within a coding sequence, can be of obvious benefit when it is associated with the expression of functional product(s). In the current work, the length of the G-tract within the first upstream gene of two pilus biosynthesis gene clusters (*bbr_0113-0115* and *bbr_1889-1886*) is shown to be crucial for functional assembly of the corresponding sortase-dependent pilus cluster. Though we do not provide direct evidence for production of the pilus proteins Bbr_0113ab and Bbr_1889ab, we believe that the observation by EM of surface exposed pilus-like structures for the IN-frame strains UCC2003-113 and UCC2003-1889 is due to the genetically modification of the nt length of the G-tract within the coding sequence of the first gene of each operon. This observation indeed provides compelling evidence for action pilus expression. The correct genotype of the G-tract is an important constraint for the expression of any downstream located gene, therefore highlighting that replication slippage at a G-tract is indeed an important mechanism for expression of the two gene clusters. The first gene of each cluster contains a G-tract whose length determines if translation initiated within the first ORF (*bbr_0113a* or *bbr_1889a*) will continue in the second ORF (*bbr_0113b* or *bbr_1889b*), (Fig. 2, A&B). Because of the lack of active translational initiation in the second ORF, productive replication slippage to generate the fusion products (Bbr_0113ab or Bbr_1889ab) is also important for expression of the downstream genes, including a dedicated sortase-encoding gene (Fig. 1). Indeed, in the absence of adequate slippage, transcription polarity can occur within *bbr_0113b* or *bbr_1189b* due to uncoupling of translation and transcription inherent to translation termination at the 3’ stop codon of the first ORF (eg. *bbr_0113a* or *bbr_1889a*)^40^.

### Authenticity of G-tract sequences

Accurate assessment of the number of bases at a homopolymeric sequence, especially at a G-tract is challenging because in vitro DNA polymerase reactions used in slippage detection are also sensitive to slippage. Here, G/C tract length was determined using Maximum Depth Sequencing (MDS)^37^ aimed at excluding errors introduced during PCR and sequencing procedures. Surprisingly, the distribution of G/C tract length, and corresponding frequency, can differ significantly from the annotated sequences (NC_020517.1). This highlights the need for caution when considering the reality of annotation of homopolymeric G-tracts present in sequence databases, with potential significance for population heterogeneity of their nt lengths at specific genomic loci. Such plasticity of nt length is also supported by our experiments as one single purified bacterial colony was used in our in vitro culture conditions. Another challenge with G-tracts is their intrinsic capability for structure formation, especially G-quadruplex structure^43^. This could be the reason for the limitation of Illumina NGS sequencing of some G-tract templates resulting in the “G’s end” phenomenon (Fig. 5). This is presumably also relevant to errors that may occur during chemical synthesis of oligonucleotides containing a G-tract.

### Regulation of replication slippage or selection of phenotype or both?

Our results show a dramatic change of slippage pattern for *bbr_0113* and *bbr_1889* sortase-dependent pili after passage of UCC2003 within the gastrointestinal environment of mice. Mechanical aspects of slippage directionality resulting in base(s) addition versus base(s) omission) has been shown for some polymerase enzymes to be related to a backward or forward re-alignment of the 3’ end of the nascent transcript with respect to its template, following by incorporation of the cognate substrate specified at the re-aligned template base position^44^. Productive slippage at *bbr_0113* and at *bbr_1889* corresponds to (−1+3n, with n integer) slippage events, with the preponderant event being the omission of a single base. Such directionality may be relevant to potential stimulation of functionally productive slippage by the relative concentration of the cognate substrate specified by the slippage-prone motif and the template base 3’ adjacent to that motif. This was shown for various slippage-prone motifs of divergent polymerases, including RNA polymerases and Reverse Transcriptases^45–47^. Because dedicated polymerases are involved in the synthesis of the leading and lagging DNA strand at the replication fork, it is also plausible that efficiency of slippage is more prevalent in one of the two newly synthesized DNA strand (SI Discussion). With DNA polymerases, small nucleotide pool imbalances have a major impact on cancer disease related to polymerase fidelity^48,49^. Interestingly, the nucleotide pool was also shown to influence G-quadruplex structure with perturbation on transcription^50^. Therefore, it is possible that the environmental condition of the gastrointestinal tract affects the intracellular nucleotide pool of UCC2003, and thereby the InDel distribution at *bbr_0113* and *bbr_1889* loci. With the replication process being semi-conservative, the InDel generated in the neo-synthesized DNA strand would ultimately generate a “bulge” at the corresponding base(s) pairing position with the original DNA template strand. Such a “bulge” is the target for universal mismatch repair system^51,52^. Therefore, regulation of InDel generation at *bbr_0113* and *bbr_1889* loci could influence the DNA repair system. This is relevant to the maintenance of the InDel within the newly synthesized chromosomal DNA strand when considering the semi-conservative process of replication (see SI Discussion and Figure S2).

While mechanical aspects of replication-slippage are important, we consider that the enrichment of ‘ON’ slippage-derived sequence found in vivo (Fig. 7) is likely explained by positive selection for the phenotype associated with production of sortase-dependent pili at the surface of the bacteria. While such pilus expression could trigger the immune system^53,54^ and so contribute to decrease their abundance, surface-exposed appendages may also be important for retention within the gut^55^. Such an in vivo/in vitro selection for particular variants is well observed in a number of host-adapted pathogens encoding genes which undergo similar phase-variation events^56,57^. Therefore, enrichment of productive slippage at *bbr_0113* and *bbr_1889* is expected to improve bacterial attachment within (certain locations within) the murine intestinal tract and so limit their elimination by defecation. This could ultimately favor the multiplication of clusters of attached bacterial having a ‘pilus-positive’ phenotype, and so enrich the abundance of the corresponding G-tract genotype in the bacterial population. Potential selection of the pilus-positive phenotype is supported by the enrichment of a genotype corresponding to *bbr_0113ab*, or *bbr_1889ab*, resulting from the fusion of two ORFs.

Broadening the scope of replication slippage utilization beyond *B. breve* UCC2003 is the presence of similar G-tract sequences present in other *Bifidobacterium* strains, including potential for combination of multiple phenotypes for some strains. With replication slippage, UCC2003 can exhibit at least two phenotypes for each of the two sortase-dependent pili. With a productive InDel, an “ON” phenotype corresponds to pilus expression, and an “OFF” phenotype to no or a functionally unproductive, InDel. Therefore, 4 relevant combinations of phenotypes could be produced within the UCC2003 population (No expression of the two sortase-dependent pili, expression of just Bbr_0113ab, expression of just Bbr_1889ab, or expression of both Bbr_0113ab and Bbr_1889ab). While being outside the scope of this manuscript, we have also identified other relevant G-tracts in UCC2003, some of them being intragenic or present within the spacer sequences of promoter sequences. Taken together, productive replication slippage within *B. breve* UCC2003 seems to be an important contributor for microdiversity generation of the UCC2003 population and so could be an important mechanism for its colonization and maintenance within the human gastrointestinal tract.

## Conclusions

1- Bioinformatic analysis show conserved intragenic G-tract sequences within the coding sequence of the sortase-dependent pilus biosynthesis genes among members of the *Bifidobacterium* genus.

2- A fraction of the population of *Bifidobacterium* possess a genotype of nucleotide G-tract length which would ultimately cause the expression of sortase-dependent pili, and perform its presumed function for epithelial cell attachment.

3- For the first time we show that replication slippage at a G-tract seems to be subject to in vivo modulation generating an enrichment of bacteria which would ultimately express sortase-dependent pili.

4- This study suggests that microdiversity generation derived by replication slippage is not a stochastic phenomenon, but that co-evolution between *Bifidobacterium* and its host has selected the G-tract sequence as a slippery prone sequence, not only to mediate a range of G tract lengths, but possibly underlying novel, yet unknown microbe-host interactions.

## Supplementary Information

Supplementary Information 1.

Supplementary Tables.

Supplementary Figure S1.

Supplementary Figure S2.

SupplementaryTable S4.

Supplementary Table S5.

## Data Availability

Raw NGS reads are deposited in SRA database under the accession number PRJNA689291. The correspondence between the names of each samples indicated in this manuscript and the raw NGS reads files deposited in SRA is in SI Table S6. Supplemental Information contains Tables S1–S6, including the description of bacteria strains and plasmids (Table S5), Figures S1–S2, and SI Discussion.

## Abbreviations

ORFs: Open Reading Frames
MDS: Maximum Depth Sequencing
LB: Luria Bertani broth
RCM: Reinforced Clostridial Medium
RCA: Reinforced Clostridial Agar
MRS: De Man Rogosa and Sharpe Medium
mMRS: Modified de Man Rogosa and Sharpe Medium
TET: Tetracycline
Em: Erythromycin
Cm: Chloramphenicol
Km: Kanamycin
X-gal: 5-Bromo-4-chloro-3-indolyl-β-D-galactopyranoside
IPTG: Isopropyl-β-D-1-thiogalactopyranoside
PCR: Polymerase Chain Reaction
PBS: Phosphate-Buffered Saline
CDS: Coding sequence
NGS: Next Generation Sequencing
PAGE: Polyacrylamide Gel Electrophoresis
HMOs: Human Milk Oligosaccharides
LNT: Lacto-N-tetraose
LNnT: Lacto-N-neotetraose
