# Supplementary material for: Maximum depth sequencing reveals an ON/OFF replication slippage switch and apparent in vivo selection for bifidobacterial pilus expression
Source: Sci Rep. 2022 Jun 10;12:9576. doi: 10.1038/s41598-022-13668-2 (PMC9187656; doi:10.1038/s41598-022-13668-2)
Supplement: Supplementary file 1 — Supplementary Information 1. [file 41598_2022_13668_MOESM1_ESM.docx]

Maximum Depth Sequencing reveals an ON/OFF replication slippage switch and apparent in vivo selection for *bifidobacterial* pilus expression.

Christophe Penno^a,1^, Mary O’Connell Motherway^a,2,3^, Yuan Fu^4^, Virag Sharma^5^, Fiona Crispie^3,6^, Paul D. Cotter^3,6^, Benoit Houeix^7^, Lokesh Joshi^7^, Francesca Bottacini^8^, Aoife O’Dwyer^3^, Gary Loughran^9^, John F. Atkins^2,9^, Douwe van Sinderen^2,3,^*

^1^ECOBIO, Université Rennes 1, Rennes, 35042, France

^2^School of Microbiology, University College Cork, Cork, T12 YT57, Ireland.

^3^APC microbiome Ireland, University College Cork, Cork, T12 YT57, Ireland.

^4^IBERS, Aberystwyth University, UK.

^5^Department of Chemical Sciences, University of Limerick.

^6^Teagasc Food Research Centre, Moorepark, Fermoy, Co. Cork P61 C996, Ireland.

^7^National University of Ireland, Galway, Ireland.

^8^Department of Biological Sciences, Munster Technological University, Cork, Ireland.

^9^School of Biochemistry, University College Cork, Cork, T12 YT57, Ireland.

^a^Christophe Penno and Mary O’Connell Motherway should be considered joint first author.

* To whom correspondence should be addressed. Tel: +353 21 4901365; Fax: +353 21 4903101; Email: d.vansinderen@ucc.ie

Address: Douwe van Sinderen, APC Microbiome Ireland, University College Cork, Cork, Ireland

**SUPPLEMENTARY DATA**

**SUPPLEMENTARY DISCUSSION**

**Indel generation and DNA correction system.**

An intriguing aspect of replication slippage is the maintenance of the InDel in the newly synthesized chromosomal DNA strand when considering the semi-conservative process of replication. Such maintenance could also be an important contributor to the efficacy of slippage. With specific one base addition in one of the newly synthetized DNA strands, this should create a bulge, with that base being in an extra-helical position within the DNA double helix due to the absence of base pairing at that position with the complementary (template) DNA strand (SI Figure S2, C & E). Conversely, with specific one base omission in the newly synthetized DNA strand, this should create an extra-helical base at the counterpart base position of the complementary (template) strand (SI Figure S2, B & D). Propagation of the corresponding slippage event in the next bacterial generation means that the bulge is not corrected by the specialized correction system to remove DNA error, and it does not inhibit subsequent replication of the chromosome containing such a bulge. Therefore, at the next chromosomal division and in the absence of slippage, one chromosome would have the former slippage event on both and fully complementary strand, with the other chromosome having the original G/C tract on both and fully complementary strand (SI Figure S2, F-I). Comparative analysis of the MDS result performed for the chromosomal DNA strand containing *bbr_0113* G-tract (with AatII or ZraI) and for chromosomal DNA strand containing the C-tract (with MluI) reveals that the C-tract DNA strand has a higher propensity for extra base(s). Such a base discrepancy between the two chromosomal DNA strands could correspond to the remaining proportion of a bulge (extra-helical cytosine base) not yet corrected by the bacterial DNA repair system. This provides evidence for two possibilities: On the one hand, addition of base C could be more preponderant during the synthesis of C-tract at the 3’ end of the neo-synthesized lagging strand (and so the template has the G-tract), (SI Figure S2, C); on the other hand, omission of base G could be more preponderant during the synthesis of the G-tract at the 3’ end of the neo-synthesized leading strand (and so the DNA template has C), (SI Figure S2,D). This also points out to the source of slippage events derived by particular polymerases, some being dedicated to the synthesis of either the leading or the lagging strand, with the latter involving more than one polymerase, including an RNA polymerase.

**Chromosomal DNA lagging strand, or leading strand or both as the main contributor of replication slippage events.**

While the authenticity of UCC2003 DNA polymerase slippage on G-tracts of the assessed pilus pilus loci is evident, consideration about the source(s) of InDel generated during the synthesis of transcript at the replication fork is merited. Replication of the chromosome occurs by a semi-conservative process where each chromosomal DNA strand is used as a template for the synthesis of a corresponding complementary DNA strand. Because synthesis of nucleic acid occurs in a 5’ to 3’ direction specified by a template orientated in a 3’ to 5’ direction, continuous synthesis of the complementary chromosomal strand (leading strand) occurs only when the progression of the replication fork takes place on a template orientated 3’ to 5’. In contrast, the counterpart chromosomal DNA strand template, orientated 5’ to 3’, undergoes discontinuous DNA synthesis of the complementary chromosomal strand (lagging strand) (SI Figure S2, A).

Relevant difference between synthesis of the leading and lagging DNA strands could also represent important features for replication slippage stimulation: (1) the propensity of slippage of polymerases involved in the synthesis of the complementary DNA strand, including a dedicated RNA polymerase involved during the synthesis of the lagging strand, (2) the propensity of realignment of the 3’ end of the transcript with respect to the template, involving a difference in the number of base pairings between the transcript and the template considering the presence of Okazaki fragments involved in the synthesis of the lagging strand, (3) the topological constraint encountered by the polymerase(s) at the back edge of the replication fork (during the synthesis of the DNA lagging strand) and the front edge of the replication fork (during the synthesis of the DNA leading strand). With the synthesis of the leading strand the topological constraint would be associated with the separation of chromosomal DNA strand at the front of the replication fork.

**SI TABLES AND FIGURES LEGENDS**

**SI Table S1. MDS samples.**

**SI Table S2. G’s ends count**

**SI Table S3. Number of family sequences generated during the analysis of InDel.**

**SI Table S4. Oligonucleotides**

**SI Table S5. Plasmids and strains.**

**SI Table S6. SRA Metadata.** Correspondence between the names of each sample in this manuscript and the name of the files containing their raw NGS reads deposited in SRA database under the accession number PRJNA689291.

**Figure S1. Genetic organization of IN-frame sequence in *bbr_0113ab* and *bbr_1889b mutants*.** Case 1 and case 2 refer to the two possible recombination events, with case 2 being the expected construct used in this study where the P44 promoter is used for the expression of the IN-frame *bbr_0113ab* and *bbr_1889ab* constructs.

**Figure S2. Propagation of InDel during the synthesis of one the neo-synthesized chromosomal DNA strand.** (**A**) The semi-conservative process of replication involved each chromosomal DNA strand (black) used as template. Because nucleic acid synthesis occurs from 5’ to 3’ (arrow direction), synthesis of the DNA strand, the leading strand (red), occurs in the same direction as the progression of the replication fork. Conversely, the synthesis of the other strand, the lagging strand (blue), is discontinuous. With chromosomal loci containing a G/C tract (yellow closed rectangle) at bacteria generation N, omission or addition of base(s) can occurs during the synthesis of either the lagging or the leading strand, with consequence to create a bulge at bacteria generation N+1 within the two newly synthesized chromosome (**B** or **C**) and (**D** or **E**). By standard replication, each chromosome molecule containing such a bulge should, at generation N+2, yield two chromosomes (**F** or **G**) and (**H** or **I**) whose full base pairing of their DNA strand is restored. One of the two has the G/C sequence of the chromosome in generation N, the other has the corresponding InDel.

**SI REFERENCES**

1. Law, J., Buist, G., Haandrikman, A., Kok, J., Venema, G. and Leenhouts, K. (1995) A system to generate chromosomal mutations in *Lactococcus lactis* which allows fast analysis of targeted genes. *J. Bacteriol.* **177**(24), 7011-7018.

2. O'Connell Motherway, M., O'Driscoll, J., Fitzgerald, G.F. and van Sinderen, D. (2009) Overcoming the restriction barrier to plasmid transformation and targeted mutagenesis in *Bifidobacterium breve* UCC2003. *Microb. Biotechnol.* **2**(3), 321-332.

3. O'Connell Motherway, M., Zomer, A., Leahy, S.C., Reunanen, J., Bottacini, F., Claesson, M.J., O'Brien, F., Flynn, K., Casey, P.G., Moreno Munoz, J.A., Kearney, B., Houston, A.M, O'Mahony, C., Higgins, D.G., Shanahan, F., Palva, A., de Vos, W.M., Fitzgerald, G.F., Ventura, M., O'Toole, P.W. and van Sinderen, D. (2011) Functional genome analysis of *Bifidobacterium breve* UCC2003 reveals type IVb tight adherence (Tad) pili as an essential and conserved host-colonization factor. *Proc. Natl. Acad. Sci. U.S.A.,* **108**(27), 11217-11222.

4. Alvarez-Martin, P., O'Connell-Motherway, M., van Sinderen, D. and Mayo, B. (2007) Functional analysis of the pBC1 replicon from *Bifidobacterium catenulatum* L48. *Appl. Microb. Biotechnol*. **76**(6): 1395-1402.

5. McGrath, S., Fitzgerald, G. F., and van Sinderen, D. (2001). Improvement and optimization of two engineered phage resistance mechanisms in *Lactococcus lactis*. *Appl. Environ. Microbiol.* **67**, 608–616. doi: 10.1128/AEM.67.2.608-616.
