# Supplementary material for: Maximum depth sequencing reveals an ON/OFF replication slippage switch and apparent in vivo selection for bifidobacterial pilus expression
Source: Sci Rep. 2022 Jun 10;12:9576. doi: 10.1038/s41598-022-13668-2 (PMC9187656; doi:10.1038/s41598-022-13668-2)
Supplement: Supplementary file 3 — Supplementary Figure S1. [file 41598_2022_13668_MOESM3_ESM.ppt]

## Slide 1
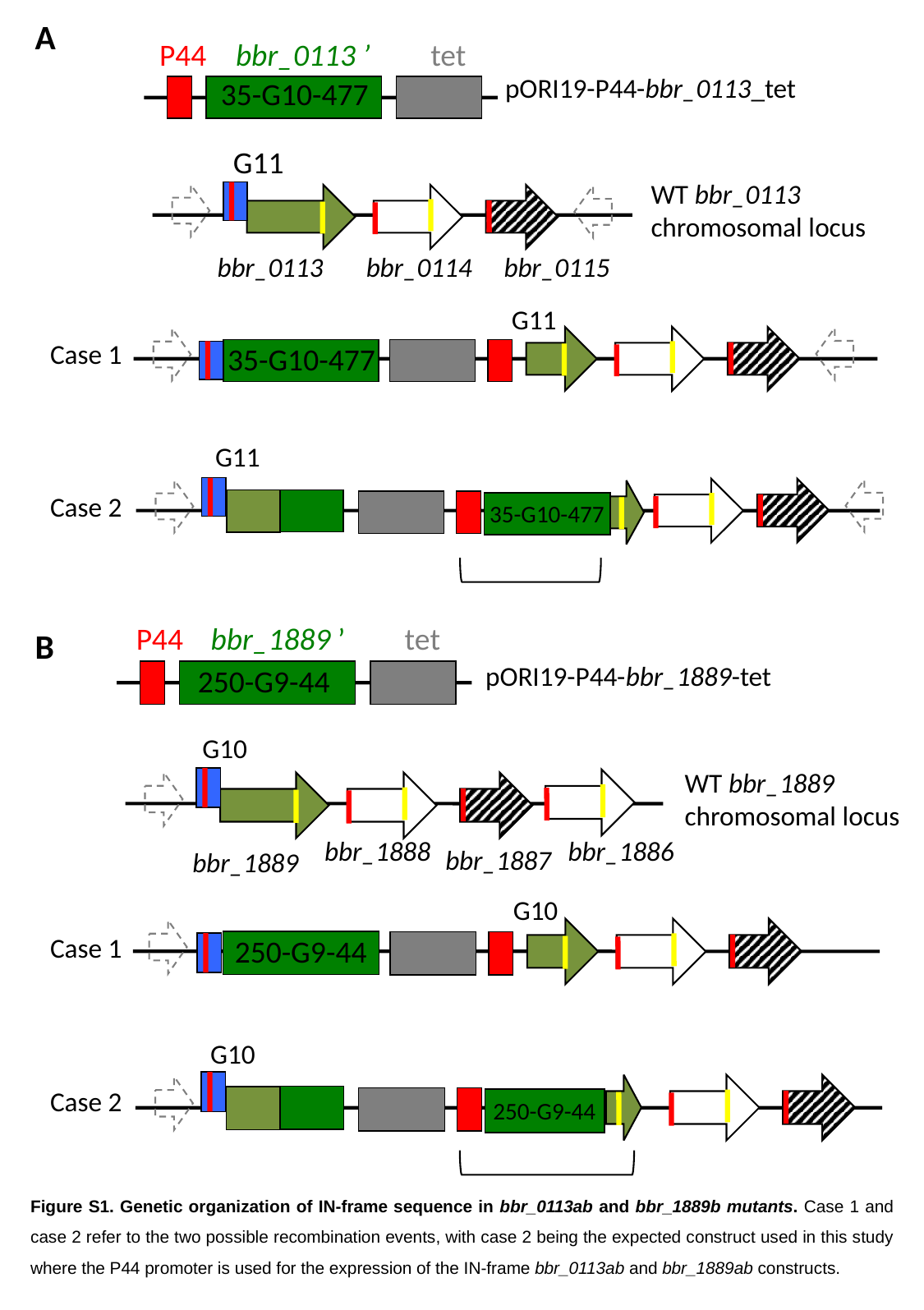

A
P44
bbr_0113 ’
tet
pORI19-P44-bbr_0113_tet
35-G10-477
G11
WT bbr_0113 chromosomal locus
bbr_0113
bbr_0114
bbr_0115
G11
Case 1
35-G10-477
G11
Case 2
35-G10-477
P44
bbr_1889 ’
tet
B
pORI19-P44-bbr_1889-tet
250-G9-44
G10
WT bbr_1889 chromosomal locus
bbr_1888
bbr_1886
bbr_1887
bbr_1889
G10
Case 1
250-G9-44
G10
Case 2
250-G9-44
Figure S1. Genetic organization of IN-frame sequence in bbr_0113ab and bbr_1889b mutants. Case 1 and case 2 refer to the two possible recombination events, with case 2 being the expected construct used in this study where the P44 promoter is used for the expression of the IN-frame bbr_0113ab and bbr_1889ab constructs.
