# Supplementary material for: Maximum depth sequencing reveals an ON/OFF replication slippage switch and apparent in vivo selection for bifidobacterial pilus expression
Source: Sci Rep. 2022 Jun 10;12:9576. doi: 10.1038/s41598-022-13668-2 (PMC9187656; doi:10.1038/s41598-022-13668-2)
Supplement: Supplementary file 4 — Supplementary Figure S2. [file 41598_2022_13668_MOESM4_ESM.pptx]

## Slide 1
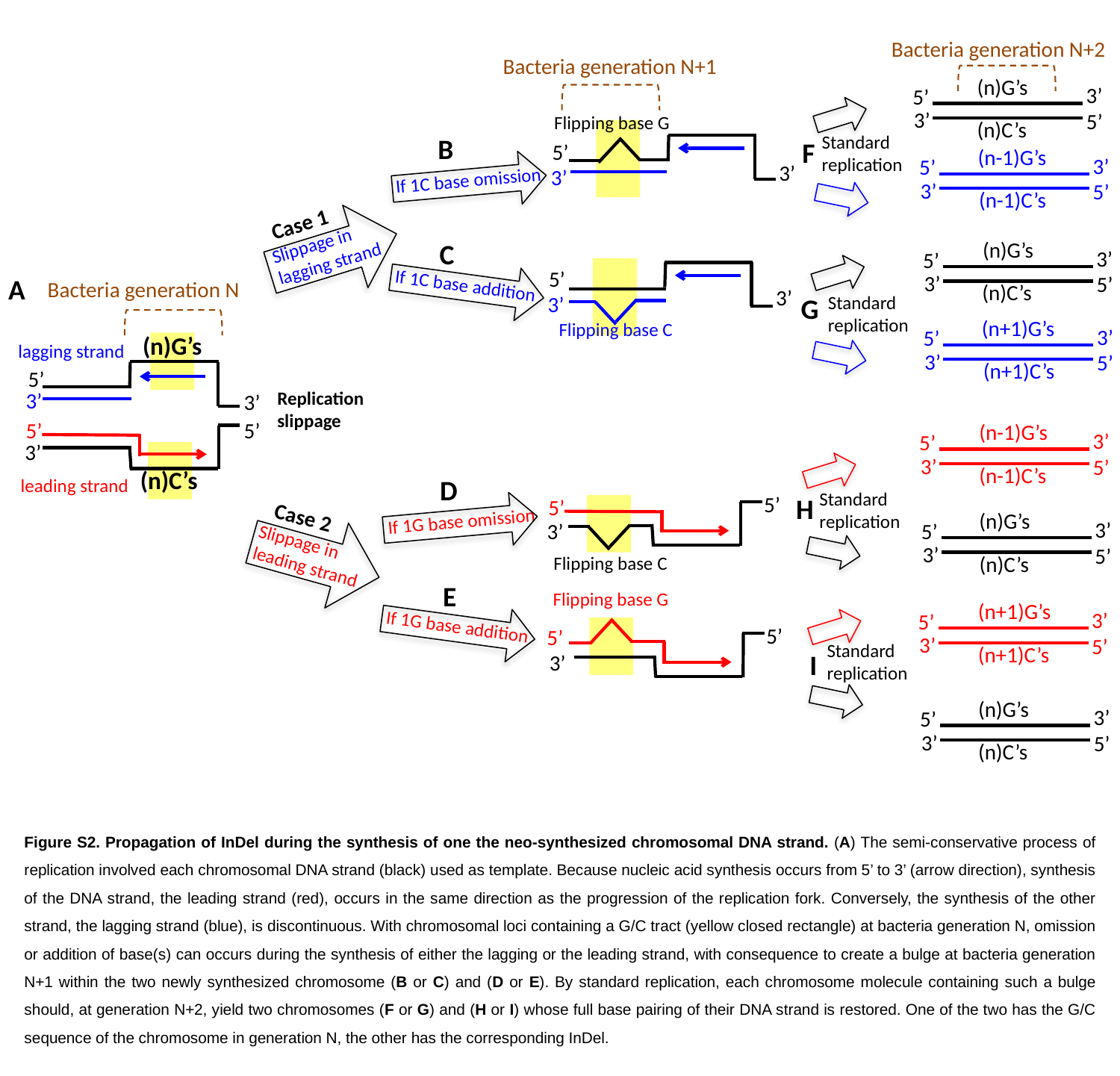

Bacteria generation N+2
Bacteria generation N+1
(n)G’s
3’
5’
3’
5’
Flipping base G
(n)C’s
Standard replication
B
F
5’
(n-1)G’s
3’
5’
3’
3’
If 1C base omission
3’
5’
(n-1)C’s
Case 1
Slippage in
lagging strand
C
(n)G’s
3’
5’
5’
3’
5’
A
Bacteria generation N
If 1C base addition
(n)C’s
3’
3’
Standard replication
G
(n+1)G’s
Flipping base C
3’
5’
(n)G’s
lagging strand
3’
5’
(n+1)C’s
5’
Replication
slippage
3’
3’
5’
5’
(n-1)G’s
3’
5’
3’
3’
5’
(n-1)C’s
(n)C’s
D
leading strand
Standard replication
5’
H
5’
If 1G base omission
Case 2
(n)G’s
3’
5’
3’
3’
5’
Slippage in
leading strand
(n)C’s
Flipping base C
E
Flipping base G
(n+1)G’s
3’
5’
If 1G base addition
5’
5’
3’
5’
Standard replication
(n+1)C’s
I
3’
(n)G’s
3’
5’
3’
5’
(n)C’s
Figure S2. Propagation of InDel during the synthesis of one the neo-synthesized chromosomal DNA strand. (A) The semi-conservative process of replication involved each chromosomal DNA strand (black) used as template. Because nucleic acid synthesis occurs from 5’ to 3’ (arrow direction), synthesis of the DNA strand, the leading strand (red), occurs in the same direction as the progression of the replication fork. Conversely, the synthesis of the other strand, the lagging strand (blue), is discontinuous. With chromosomal loci containing a G/C tract (yellow closed rectangle) at bacteria generation N, omission or addition of base(s) can occurs during the synthesis of either the lagging or the leading strand, with consequence to create a bulge at bacteria generation N+1 within the two newly synthesized chromosome (B or C) and (D or E). By standard replication, each chromosome molecule containing such a bulge should, at generation N+2, yield two chromosomes (F or G) and (H or I) whose full base pairing of their DNA strand is restored. One of the two has the G/C sequence of the chromosome in generation N, the other has the corresponding InDel.
