# Supplementary material for: Maximum depth sequencing reveals an ON/OFF replication slippage switch and apparent in vivo selection for bifidobacterial pilus expression
Source: Sci Rep. 2022 Jun 10;12:9576. doi: 10.1038/s41598-022-13668-2 (PMC9187656; doi:10.1038/s41598-022-13668-2)
Supplement: Supplementary file 5 — SupplementaryTable S4. [file 41598_2022_13668_MOESM5_ESM.docx]

**SI Table S4. Oligonucleotide primers used in this study**

***Purpose*** ***Primer*** ***Sequence***

**Construction UCC2003 in-frame sortase dependent pili mutants**

***Amplification of fragment of bbr_0113*** 113F CTGCATCCATGGGAGACGAGGAAAGCATGATGATAGCGAGAAGTTCCGGGGGGGGGGCAGCGAAATTCAAG

113R CTGCGATCTAGACATCAGCGGTGGAAGTGG

***Amplification of fragment of bbr_1889*** 1889F CTGCATCCATGGGATCGTATGCAGTAACCTC

1889R CTATGCTCTAGACAACGTTGACATTGCGAGCAGAAGGAGCAGCTACGACAGTATTACCCCCCCCCGAAATTACGGCGTTAGGAGAGC

***Amplification of P44-113 or P44-1889*** P44forward TGCGGAAAGCTTGTTAGTTGAAGAAGGTTTTTATATTAC

***Confirmation of site specific homologous*** P44 confirm GAGATAATGCCGACTGTAC

***recombination*** 113confirm GCGTCTTGGTTCTGAGTATTC

1889confirm GTCTGTCGCACCATTTGAG

***Amplification of tetW*** tetWFw TCAGCTGTCGACATGCTCATGTACGGTAAGGAAGCA

tetWRv GCGACGGTCGACCATAACTTCTGATTGTTGCCG

**Maximum Depth Sequencing NGS analysis**

***MDS Forward adapter amplifier*** F286 TCGTCGGCAGCGTCAGATG

***MDS Reverse adapter amplifier*** R287 GTCTCGTGGGCTCGGAGATG

***MDS_****AatII* ***14nt family tag*** *bbr_0113* F285 TCGTCGGCAGCGTCAGATGTGTATAAGAGACAGNNNNNNNNNNNNNNACGTCACCAGCACGGCAGCG

***MDS PCR*** *bbr_0113* R293 GTCTCGTGGGCTCGGAGATGTGTATAAGAGACAGNNNNNNNNCGCCGGAGACGAGGAAAGC

***MDS_****AatII* ***Control*** *bbr_0113* (PAGE purified) F449

CGCCGGAGACGAGGAAAGCATGATGATAGCGAGAAGTTCCgggggggggggCAGCGAAATTCAAGGCTGCCGCCGCTGCCCTGCTTGCCGCTGCCGTGCTGGTGACGT

***MDS_****ZraI* ***14nt family tag*** *bbr_0113* F288 TCGTCGGCAGCGTCAGATGTGTATAAGAGACAGNNNNNNNNNNNNNNGTCACCAGCACGGCAGCGGCTT

***MDS_****MluI* ***14nt family tag*** *bbr_0113* F289 TCGTCGGCAGCGTCAGATGTGTATAAGAGACAGNNNNNNNNNNNNNNTTAGACGACGGTTC**GCC**GGAG

***MDS PCR*** *bbr_0113* R294 GTCTCGTGGGCTCGGAGATGTGTATAAGAGACAGNNNNNNNNGCGGCAAGCAGGGCAGC

***MDS_****MluI* ***Control*** *bbr_0113* (PAGE purified) F450

GCGGCAAGCAGGGCAGCGGCGGCAGCCTTGAATTTCGCTGcccccccccccGGAACTTCTCGCTATCATCATGCTTTCCTCGT**CTCCGGCGAACCGTCGTCTA**A

***MDS_****AatII* ***14nt family tag*** *bbr_1889* F290 TCGTCGGCAGCGTCAGATGTGTATAAGAGACAGNNNNNNNNNNNNNNACGTCT*T*ATTGTCAAC**GTTGACA**TT*G*

***MDS_****ZraI* ***14nt family tag*** *bbr_1889* F291 TCGTCGGCAGCGTCAGATGTGTATAAGAGACAGNNNNNNNNNNNNNNGTCTTATTGTCAAC**GTTGACA**TTG

***MDS PCR*** *bbr_1889* R295 GTCTCGTGGGCTCGGAGATGTGTATAAGAGACAGNNNNNNNNTATGCTCAGAATATAGACAATG

***MDS_****MluI* ***14nt family tag*** *bbr_1889* F359 TCGTCGGCAGCGTCAGATGTGTATAAGAGACAGNNNNNNNNNNNNNNACGTCT*T*ATTGTCAAC

***MDS_****AatII* ***Control*** *bbr_1889* (PAGE purified) F451

TATGCTCAGAATATAGACAATGTGAGTAGCTCTCCTAACGCCGTAATTTCGGGGGGGGGGTAATACTGTCGTAGCTGCTCCTTCTGCTCG**CAATGTCAACGTTGACAATAA**GACGT
