# Supplementary material for: Maximum depth sequencing reveals an ON/OFF replication slippage switch and apparent in vivo selection for bifidobacterial pilus expression
Source: Sci Rep. 2022 Jun 10;12:9576. doi: 10.1038/s41598-022-13668-2 (PMC9187656; doi:10.1038/s41598-022-13668-2)
Supplement: Supplementary file 6 — Supplementary Table S5. [file 41598_2022_13668_MOESM6_ESM.docx]

**SI Table S5. Bacterial strains used in this study**

| **Strains and plasmids** | **Relevant features** | **Reference or source** |
| --- | --- | --- |
| **Strains** |  |  |
| *Escherichia coli* strains |  |  |
| *E. coli* EC101 | Cloning host, repA^+^ km^r^ | (1) |
| *E. coli* EC101-pNZ-M.BbrII + M.BbrIII | EC101 harbouring pNZ8048 derivative containing *bbrIIM* and *bbrIIIM*. | (2) |
|  |  |  |
|  |  |  |
|  |  |  |
| *Bifidobacterium* sp. strains |  |  |
| *B. breve* UCC2003 | Isolate from nursling stool | (3) |
| *B. breve* UCC2003-113 | tadA insertion mutant of *B. breve* UCC2003 | This study |
| *B. breve* UCC2003-1889 | tadE deletion mutant of UCC2003 | This study |
|  |  |  |
| ***Plasmids*** |  |  |
| pAM5 | pBC1-puC19-Amp^r^-Tet^r^ | (4) |
| pORI19 | Em^r^, repA^-^, ori^+^, cloning vector | (1) |
| pNZ44 | P44 promoter fusion vector Cm^r^ | (5) |
| pORI19-P44-113-tet | P44 fused fragment of *bbr_0113* (516 bp) and tetW cloned in pORI19 | This study |
| pORI19-P44-1889-tet | P44 fused fragment of *bbr_1889* (301 bp) and tetW cloned in pORI19 | This study |
